# Effectiveness of Medhasagar Rasa® in Combating Aging-Associated Mild Neurocognitive Disorder: An Open-Label, Exploratory, Interventional Clinical Trial

**DOI:** 10.7759/cureus.69561

**Published:** 2024-09-16

**Authors:** Suhas Kumar Shetty, Aswini Ramachandran, Mukesh Chawda, Sangam Narvekar, Megha Nalawade, Mohit Sharma, Rajmohan Seetharaman

**Affiliations:** 1 Department of Internal Medicine, Karnatak Lingayat Education Academy of Higher Education and Research’s Shri B. M. Kankanawadi Ayurveda Mahavidyalaya, Belagavi, IND; 2 Department of Medical Services, Solumiks Herbaceuticals Limited, Mumbai, IND; 3 Department of Medical Services, Shree Dhootapapeshwar Limited, Mumbai, IND; 4 Department of Clinical Research, Shree Dhootapapeshwar Limited, Mumbai, IND; 5 Department of Pharmacology, Mahatma Gandhi Mission Medical College and Hospital, Nerul, Mahatma Gandhi Mission Institute of Health Sciences, Navi Mumbai, IND

**Keywords:** aging-associated disorders, brief cognitive rating scale (bcrs), clinical global impression (cgi), cognitive enhancers, hamilton anxiety rating scale (ham-a), mild cognitive impairment, montreal cognitive assessment (moca), pittsburgh sleep quality index (psqi)

## Abstract

Introduction: With the rising prevalence of neurocognitive disorders (NCDs) among the aging population, particularly in conditions like mild cognitive impairment (MCI), which often precedes dementia, there remains a significant gap in effective pharmacological interventions. This has generated interest in exploring alternative therapies to manage symptoms and enhance cognitive function in the aging population. The primary objective of this study was to evaluate the effect of Medhasagar Rasa® on cognitive functions, daily functioning, and quality of life in participants with aging-associated mild neurocognitive disorder using the Montreal Cognitive Assessment (MoCA) Scale, Ayurvedic Manasabhava Scale, and Brief Cognitive Rating Scale (BCRS).

Methods: This open-label, interventional study at Karnatak Lingayat Education (KLE) Ayurveda Hospital, Belagavi, Karnataka, involved 32 screened participants, with 30 completing the study. Participants aged 50-70 years with MoCA scores of 18-25 received Medhasagar Rasa (2 tablets at bedtime, provided by M/s. Shree Dhootapapeshwar Limited, Mumbai, India) for 60 days. Assessments occurred at baseline and every 15 days until day 60.

Results: Thirty participants were recruited for the study after screening, all of whom completed the study. The median total MoCA score at baseline (visit one) was 20, which significantly improved to 25 by visit five (day 60±3) (p<0.001), indicating enhanced cognitive performance. The BCRS scores also showed significant improvement, with the median score decreasing from 12 to 7.5 (p<0.001) over 60 days. Anxiety symptoms were significantly reduced, with Hamilton Anxiety Rating Scale (HAM-A) scores dropping from 14 to 7 (p<0.001), while the Pittsburgh Sleep Quality Index (PSQI) scores indicated improved sleep quality, reducing from 9.5 to 7 (p<0.001). The Ayurvedic Manasabhava Scale also demonstrated a significant reduction in intensity (14 to 6; p<0.001) and frequency (13.5 to 6; p<0.001). Clinical Global Impression (CGI) scores showed stable illness severity, sustained global improvement, and consistent therapeutic efficacy. No adverse events were reported, and vital parameters remained normal throughout the study. Compliance with the medication was over 80%, and no significant changes were observed in laboratory values.

Conclusion: Medhasagar Rasa effectively enhanced cognitive functions and alleviated anxiety and sleep disturbances in aging-related mild neurocognitive disorder, offering a promising therapeutic option.

## Introduction

Neurocognitive disorders (NCDs) become increasingly prevalent with age. NCD is a broad term encompassing various diagnoses that manifest as cognitive function impairment and range from subtle, mild cognitive impairments to severe, disabling dementias [1]. Diagnostic and Statistical Manual-5 (DSM-5) has classified acquired neurocognitive disorders of all age groups under three major headings: delirium, major NCD, and mild NCD. The prevalence of mild and major NCDs, weighted to the population, is 17.6% and 7.2%, respectively, in India. Given an estimated 138 million adults over 60 years of age in India, these estimates suggest approximately 24 million and 9.9 million older adults in India are living with mild and major neurocognitive disorders, respectively [2]. The most prevalent types of NCDs are Alzheimer's disease (70% of NCDs), vascular disease (10%-20% of NCDs), and Lewy bodies (3%-7% of NCDs). It is estimated that 50% of the NCD population has mixed dementia, which usually includes both Alzheimer's and a vascular presentation [3].

Currently, there is insufficient evidence to support specific interventions for cognitive support in mild cognitive impairment (MCI) [4]. While treatments aimed at alleviating symptoms of MCI can offer benefits to patients, research is primarily focused on identifying interventions that can slow the progression of cognitive decline. However, no study to date has demonstrated the ability to delay the transition from MCI to dementia. Potential treatments being explored for MCI include acetylcholinesterase inhibitors, glutamate receptor modulators, antioxidants, anti-inflammatory agents, nootropics, immunomodulators, such as amyloid antibodies, secretase inhibitors, and Ginkgo biloba-derived products like EGb761® [5]. Cholinesterase inhibitors, commonly prescribed for Alzheimer's disease, are not recommended for mild neurocognitive disorders due to a lack of demonstrated efficacy [6-7]. Despite these efforts, no medications have shown conclusive effectiveness, leaving non-pharmacological strategies, such as aerobic exercise, cognitive stimulation, and social engagement, as the most reliable ways to mitigate further cognitive decline [8]. Additionally, collaborative approaches like art therapy, in partnership with healthcare providers, have been reported to reduce depression in individuals with mild NCDs [9]. Nevertheless, the absence of consistently effective pharmacological interventions highlights the urgent need for further research into alternative therapeutic strategies.

Ayurved Rasayana and Integrated Yoga interventions have shown the potential to enhance cognitive abilities in elderly individuals with mild NCDs, improving learning, attention, processing speed, and working memory [10]. Nootropics, or cognitive enhancers, are referred to as Medhya Rasayana in Ayurved. Medhasagar Rasa®, a proprietary Medhya Rasayana, combines classical herbomineral formulations and herbs to support brain functions and reduce stress. It includes Mahakalyan Vati (*Suvarnayukta*), Smrutisagar Rasa, Rajata Bhasma (processed silver), and Abhraka Bhasma (processed mica), as well as Medhya Rasayana herbs like Mandookaparni (*Centella asiatica*), Yashti (*Glycyrrhiza glabra*), Shankhapushpi (*Convolvulus pluricaulis*), Guduchi (*Tinospora cordifolia*), and additional herbs like Ashvagandha (*Withania somnifera*) and Haridra (*Curcuma longa*) [11-18]. Suvarna Bhasma (processed gold) in Mahakalyan Vati, enhances cognitive functions and has anxiolytic effects [19]. Suvarna Bhasma’s neuroprotective properties are documented in a model of Parkinson’s disease [20]. Smrutisagar Rasa is processed with Vacha (*Acorus calamus*), Brahmi (*Bacopa monnieri*), and Jyotishmati (*Celastrus paniculatus*), which help prevent memory impairment and oxidative stress [12, 21-23]. Medhya Rasayana herbs are renowned for their memory-enhancing and neuroprotective actions [15-16]. Ashvagandha (*Withania somnifera*) and Haridra (*Curcuma longa*) are known to prevent amyloid-β plaque build-up in the brain [17-18]. Medhasagar Rasa therefore shows potential in addressing cognitive decline in MCI through its combination of neuroprotective and memory-enhancing ingredients, offering a promising alternative for improving cognitive function and slowing progression in aging individuals.

The primary objective of this study was to assess the impact of Medhasagar Rasa on cognitive functions, daily functioning, and overall quality of life in elderly individuals with aging-associated mild NCDs. These were evaluated using the Montreal Cognitive Assessment (MoCA) Scale, Ayurvedic Manasabhava Scale, and Brief Cognitive Rating Scale (BCRS). Secondary objectives included evaluating the effect of Medhasagar Rasa on disease severity, anxiety, and sleep quality, using the Clinical Global Impression (CGI) Scale, Hamilton Anxiety Rating Scale (HAM-A), and Pittsburgh Sleep Quality Index (PSQI), respectively. Additionally, the study aimed to assess the safety of Medhasagar Rasa by monitoring liver function tests (serum glutamic oxaloacetic transaminase (SGOT) and serum glutamic pyruvic transaminase (SGPT)) and renal function tests (serum creatinine and blood urea nitrogen (BUN)).

## Materials and methods

Study design and approvals

This open-label, interventional study was conducted as a single-arm exploratory trial, with no control arm included, given the study's focus on evaluating preliminary efficacy and safety outcomes of Medhasagar Rasa. The study was approved by the Institutional Ethics Committee for Research on Human Subjects at Karnatak Lingayat Education Academy of Higher Education and Research’s (KAHER’s) Shri B. M. K. Ayurveda Mahavidyalaya, Belagavi, Karnataka, under review number KLE/BMK/MRC/956/22 on 18.08.2022. The study was initiated following Ethics Committee approval and Clinical Trials Registry - India (CTRI) registration on 08.12.2022 (CTRI registration no.: CTRI/2022/12/047965). Recruitment occurred over six months, with the first participant enrolled on 07.02.2023 and the last participant’s final visit on 15.09.2023.

Study interventions

Medhasagar Rasa, a proprietary herbomineral ayurvedic formulation approved by the Indian Government's Ministry of Ayurveda, Yoga, Naturopathy, Unani, Siddha, and Homeopathy (AYUSH) under the indication of Medhya (nootropic) on 18th May 2024, has been marketed in India for the past three years. Participants received Medhasagar Rasa, provided by M/s. Shree Dhootapapeshwar Limited, Mumbai, India, manufactured according to the good manufacturing practice (GMP) standards by Om Pharmaceuticals Limited, Bengaluru, Karnataka, India. Each participant was given two blister packs (30 tablets per pack) at visit one (day zero) and one blister pack at visits two (day 15±3) and three (day 30±3). The dosage was two tablets at bedtime with lukewarm water for 60 days. The composition of Medhasagar Rasa includes 10 mg of Mahakalyan Vati (Suvarnayukta), 100 mg of Smrutisagar Rasa, 5 mg of Rajata Bhasma, 5 mg of Abhraka Bhasma, and extracts from 100 mg each of Mandookaparni (*Centella asiatica*), Yashti (*Glycyrrhiza glabra*), Ashvagandha (*Withania somnifera*), Haridra (*Curcuma longa*), Shankhapushpi (*Convolvulus pluricaulis*), and Guduchi (*Tinospora cordifolia*).

Selection criteria

Participants aged 50-70 years, of either sex, diagnosed with mild NCDs as per DSM-V criteria, and scoring 18 to 25 on the MoCA Scale, were eligible. Exclusion criteria included other NCDs, neuropsychiatric disorders, severe or uncontrolled diabetes, hypertension, thyroid disorders, clinically relevant depression (Cornell Scale for Depression in Dementia >10), and the use of psychotropic or neurotropic medications within the past four weeks.

Study variables and endpoints

In this study, primary efficacy variables included the MoCA Scale, the Manasabhava Scale, and the BCRS scores. The MoCA Scale evaluated cognitive function across several domains, such as attention, memory, language, and executive functions. The Manasabhava Scale provided insights into both cognitive and psychological impacts, while the BCRS assessed the severity of cognitive impairment and its effect on daily living. These primary efficacy variables were analyzed at multiple time points: baseline (day 0), day 15±3, day 30±3, day 45±3, and day 60±3 to track changes in cognitive function and the overall impact of the intervention.

Secondary efficacy variables included the CGI Scale, the HAM-A, and the PSQI scores. These measures assessed overall clinical status, anxiety severity, and sleep quality, respectively. Safety variables such as liver function tests (SGOT and SGPT) and renal function tests (serum creatinine and BUN) were evaluated at baseline and at the end of the study to monitor any potential adverse effects of the intervention. The visit-wise schedule for outcome measurements is detailed in Table 1.

**Table 1:** Visit-wise schedule of study participants

No.	Procedures	Visit 1 (Day 0)	Visit 2 (Day 15±3)	Visit 3 (Day 30±3)	Visit 4 (Day 45±3)	Visit 5 (Day 60±3)
1	Screening and informed consent	✓	-	-	-	-
2	Medical history and demographic information	✓	-	-	-	-
3	Physical examination (vitals and systemic examination), height (cm), body weight (kg), and BMI (kg/m^2^).	✓	✓	✓	✓	✓
4	Assessment					
i	Montreal Cognitive Assessment (MoCA) Scale score	✓	✓	✓	✓	✓
ii	Manasabhava Scale score	✓	✓	✓	✓	✓
iii	Brief Cognitive Rating Scale (BCRS) score	✓	✓	✓	✓	✓
iv	Clinical Global Impression (CGI) Scale score	✓	✓	✓	✓	✓
v	Hamilton Anxiety Rating Scale (HAM-A) score	✓	✓	✓	✓	✓
vi	Pittsburg Sleep Quality Index (PSQI) score	✓	✓	✓	✓	✓
5	Investigations					
i	ECG	✓	-	-	-	-
ii	Fasting blood sugar and postprandial blood sugar	✓	-	-	-	✓
iii	Liver function tests (SGOT and SGPT)	✓	-	-	-	✓
iv	Renal function tests (serum creatinine and BUN)	✓	-	-	-	✓
6	Lifestyle, diet, and exercise advice	✓	✓	✓	✓	✓
7	Medication dispensing	✓	✓	✓	-	-
8	Medication compliance	-	✓	✓	✓	✓
9	Recording adverse events	✓	✓	✓	✓	✓

Sample size and data analysis

The sample size was set at 30 participants, with no formal calculation due to the exploratory nature of the study. However, a post-study power analysis was conducted to evaluate whether the study was adequately powered. Data were analyzed using IBM SPSS Statistics for Windows, Version 21, (Released 2012; IBM Corp., Armonk, New York, United States) software with descriptive statistics, expressed as percentages and mean ± SD/median (IQR). The normality of data was assessed using the Wilk-Shapiro test. Comparisons of data at two intervals were performed using a paired t-test (for normally distributed data) or Wilcoxon signed-rank test (for non-normally distributed data), with a significance level set at p<0.05.

## Results

**Figure 1:**
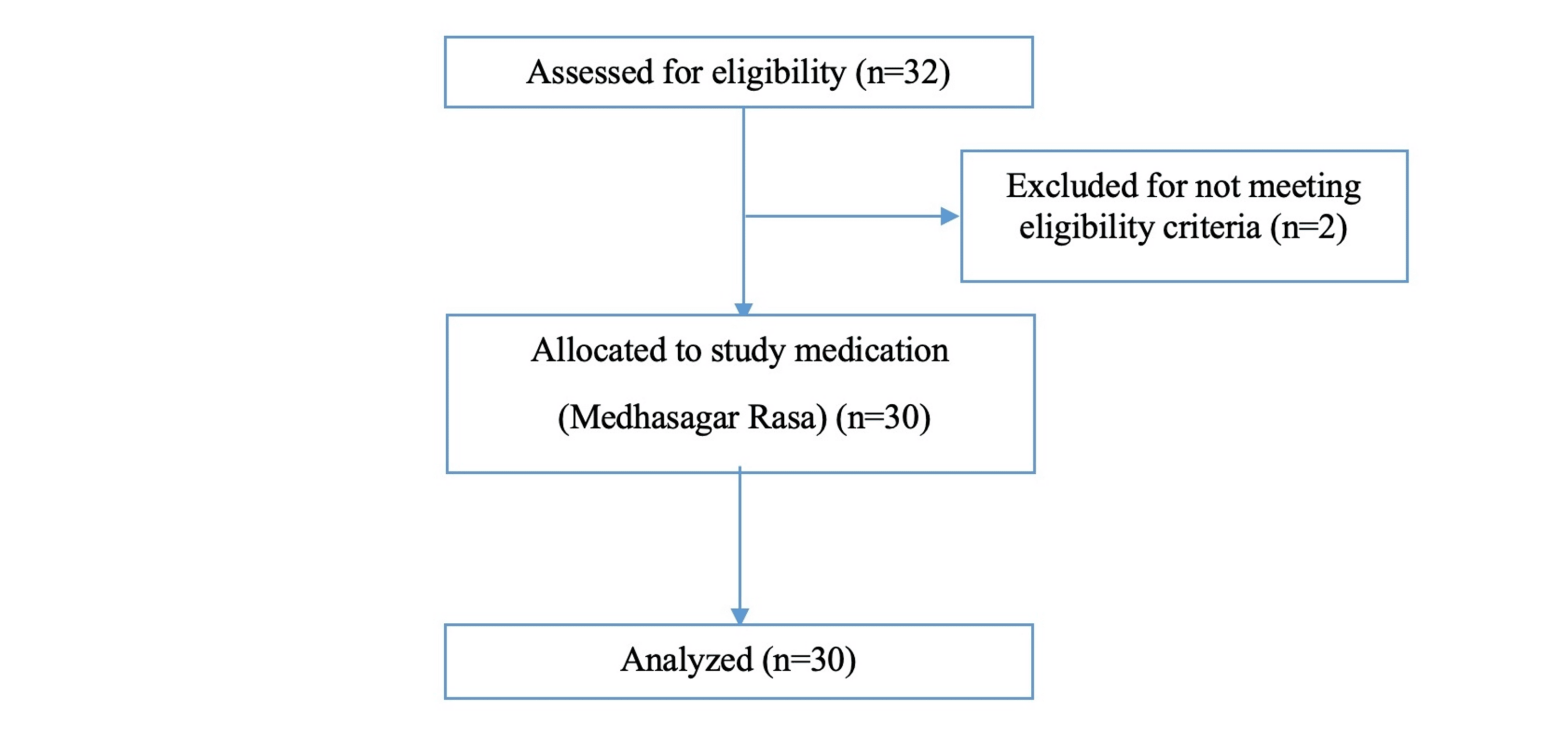
Consort flow diagram of study participants

Thirty-two participants were screened for eligibility to participate in the study following written informed consent, out of which, two participants were not found to be eligible. Thus, 30 participants were recruited for the study and were given the study medication, i.e., Medhasagar Rasa as per the protocol. All 30 participants recruited for the study completed the study and were evaluated at the end of the study period. A Consolidated Standards of Reporting Trials (CONSORT) flow diagram of the study participants is given below (Figure 1).

A total of 16 participants (53.30%) were females, and 14 (46.70%) participants were males. The baseline parameters like age, BMI, and fasting blood sugar (FBS) are mentioned in Table 2.

**Table 2:** Baseline parameters of study participants

Variable	Visit 1 (Baseline; Day 0) values (mean ± SD)
Age (years)	60.10 ± 6.02
BMI (kg/m^2^)	23.29 ± 3.08
Fasting blood sugar (FBS) (mg/dL)	93.90 ± 13.17

Primary endpoints: changes in total scores of the MoCA Scale, Manasabhava Scale, and BCRS at various time points

A. Changes in MoCA Scale Scores at Various Time Points

In this study, the MoCA Scale was used as a comprehensive tool to evaluate various cognitive functions essential for daily functioning. It assesses different cognitive domains: attention and concentration, executive functions, memory, language, visuoconstructional skills, conceptual thinking, calculations, and orientation [24,25]. At all five visits (visit one to visit five), the participants went through assessments for each domain, facilitating a longitudinal examination of cognitive performance.

**Figure 2:**
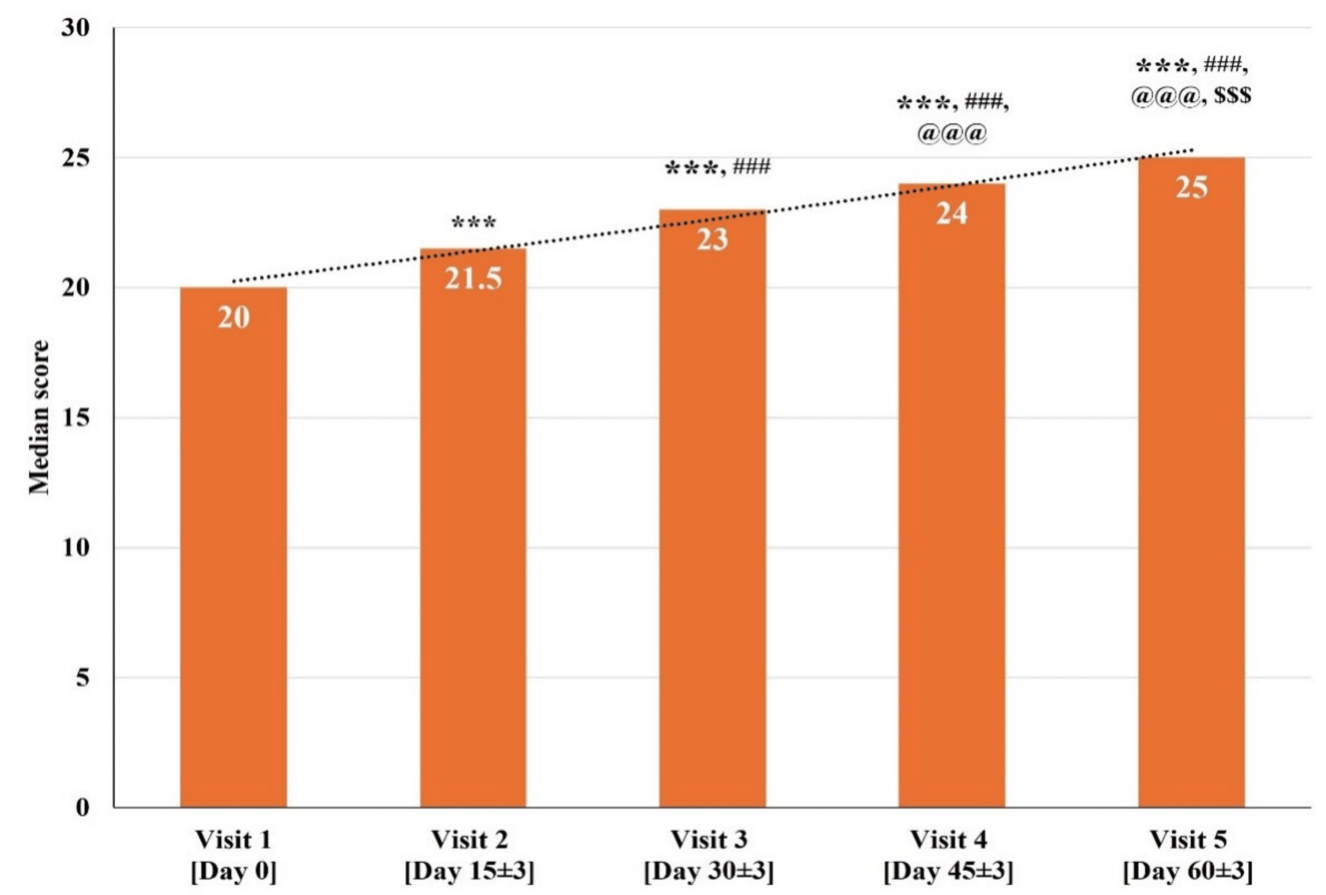
Changes in the total Montreal Cognitive Assessment (MoCA) Scale scores at various time points p<0.05 was considered significant. Results are expressed as median (IQR). Data were analyzed using the Wilcoxon signed-rank test. ^***^p<0.001 as compared to visit one (baseline; day zero), ^###^p<0.001 as compared to visit two (day 15±3), ^@@@^p<0.001 as compared to visit three (day 30±3), and^ $$$^p<0.001 as compared to visit four (day 45±3).

The total MoCA Scale scores for all 30 participants at visit one (baseline visit; day zero) were between 18 and 23. The median total MoCA Scale score significantly improved (p<0.001) on visit two (day 15±3), visit three (day 30±3), visit four (day 45±3), and visit five (day 60±3) as compared to visit one (baseline visit; day zero). There was a significant improvement (p<0.001) in the median total MoCA Scale score at visit three (day 30±3), visit four (day 45±3), and visit five (day 60±3) as compared to visit two (day 15±3). Further, a significant improvement (p<0.001) in the median total MoCA Scale score at visit four (day 45±3) and visit five (day 60±3) as compared to visit three (day 30±3) was observed. Also, a significant improvement (p<0.001) was observed in the median total MoCA Scale score at visit five (day 60±3) as compared to visit four (day 45±3) (Figure 2 and Table 3).

**Table 3:** Changes in the total Montreal Cognitive Assessment (MoCA) Scale scores at various time points p<0.05 was considered significant. Results are expressed as Median (IQR). Data were analyzed using the Wilcoxon signed-rank test. ^***^p<0.001 as compared to visit one (baseline; day zero), ^###^p<0.001 as compared to visit two (day 15±3), ^@@@^p<0.001 as compared to visit three (day 30±3), and ^$$$^p<0.001 as compared to visit four (day 45±3).

Parameter	Value	Visit one (V1) (baseline; day zero)	Visit two (V2) (day 15±3)	Visit three (V3) (day 30±3)	Visit four (V4) (day 45±3)	Visit five (V5) (day 60±3)
Total MoCA Scale score	Minimum	18.0	18.0	18.0	21.0	21.0
	Median (IQR)	20.00 (3.00)	21.50 (2.00) ***	23.00 (3.00) *** ###	24.00 (2.00) *** ### @@@	25.00 (2.00) *** ### @@@ $$$
	Maximum	23.0	25.0	25.0	27.0	27.0

The MoCA Scale score of 26 or above is considered normal [24,25]. In this study, the median total MoCA Scale score at visit one (baseline visit; day zero) was 20, which gradually improved to a median score of 25 at visit five (day 60±3). The total MoCA Scale score exhibited a significant improvement across all the visits, suggesting consistent and progressive improvement in overall cognitive performance throughout the assessment period of 60 days.

Although the domains (memory/delayed recall, attention, and orientation) of the MoCA Scale also exhibited improvement, the statistical analysis for individual domains was not feasible due to the high number of ties and small sample size.

B. Changes in Total Manasabhava Scale (Intensity) Scores at Various Time Points

In the absence of standard Ayurved scales for the assessment of neurocognitive disorders, the Manasabhava Scale commonly used by the investigators in their OPD was used in this study [26]. This scale broadly covers the domains of cognition, mood, and sleep. At all five visits (visit one to visit five), participants went through assessments based on the Manasabhava Scale.

**Figure 3:**
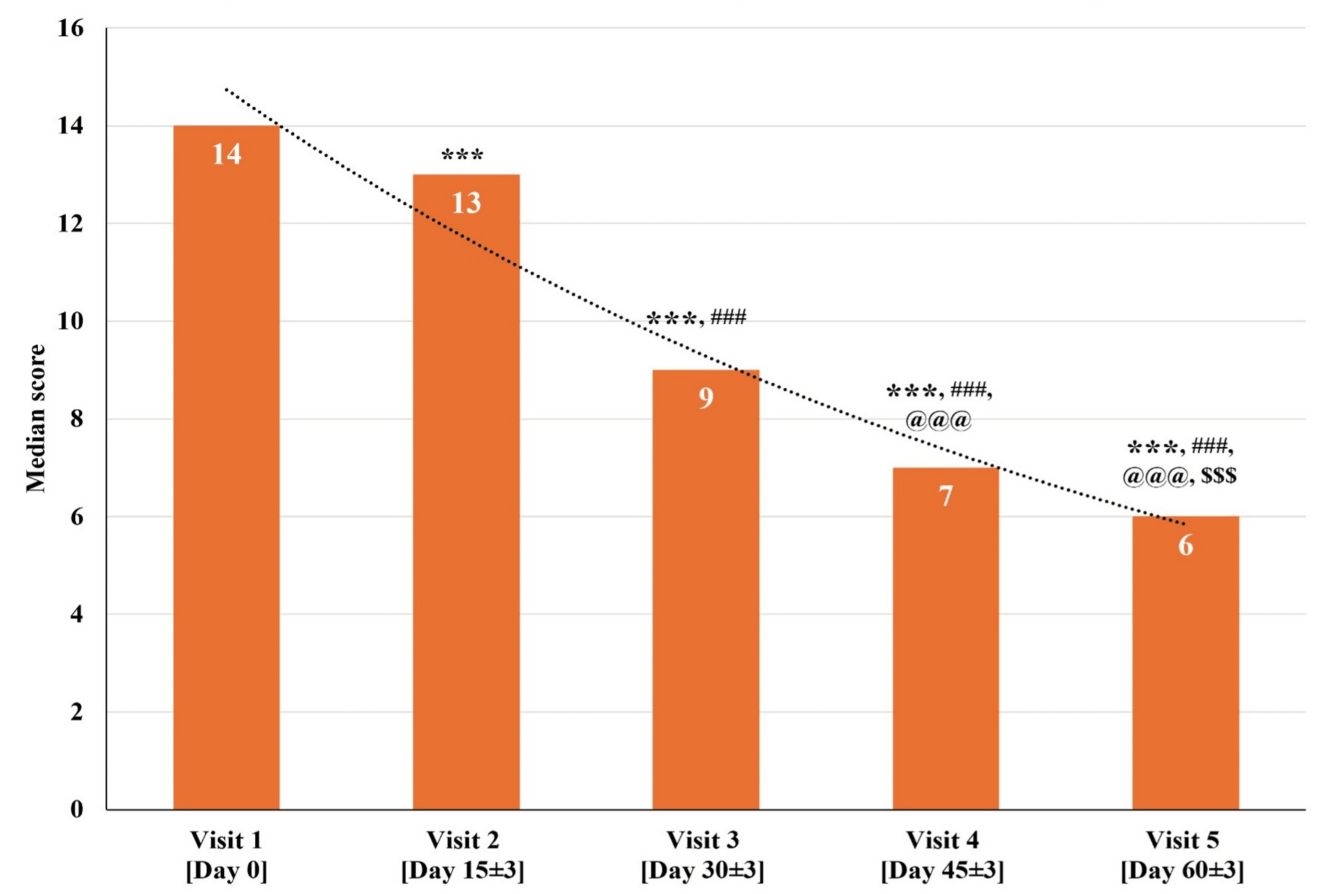
Changes in the total Manasabhava Scale (intensity) scores at various time points p<0.05 was considered significant. Results are expressed as median (IQR). Data were analyzed using the Wilcoxon signed-rank test. ^***^p<0.001 as compared to visit one (baseline; day zero), ^###^p<0.001 as compared to visit two (day 15±3), ^@@@^p<0.001 as compared to visit three (day 30±3), and ^$$$^p<0.001 as compared to visit four (day 45±3).

The total Manasabhava Scale (intensity) score for all 30 participants at visit one (baseline visit; day zero) was between 11 and 15. The median total Manasabhava Scale (intensity) score significantly reduced (p<0.001) on visit two (day 15±3), visit three (day 30±3), visit four (day 45±3), and visit five (day 60±3) as compared to visit one (baseline visit; day zero). There was a significant reduction (p<0.001) in the median total Manasabhava Scale (intensity) score at visit three (day 30±3), visit four (day 45±3), and visit five (day 60±3) as compared to visit two (day 15±3). Further, there was a significant reduction (p<0.001) in the median total Manasabhava Scale (intensity) score at visit four (day 45±3), and visit five (day 60±3) as compared to visit three (day 30±3). Also, a significant reduction (p<0.001) was observed in the median total Manasabhava Scale (intensity) score at visit five (day 60±3) as compared to visit four (day 45±3) (Figure 3 and Table 4).

**Table 4:** Changes in total Manasabhava Scale (Intensity) scores at various time points p<0.05 was considered significant. Results are expressed as Median (IQR). Data were analyzed using the Wilcoxon signed-rank test. ^***^p<0.001 as compared to visit one (baseline; day zero), ^###^p<0.001 as compared to visit two (day 15±3), ^@@@^p<0.001 as compared to visit three (day 30±3), and ^$$$^p<0.001 as compared to visit four (day 45±3).

Parameter	Value	Visit one (V1) (baseline; day zero)	Visit two (V2) (day 15±3)	Visit three (V3) (day 30±3)	Visit four (V4) (day 45±3)	Visit five (V5) (day 60±3)
Total Manasabhava Scale (intensity) score	Minimum	11.0	9.0	6.0	4.0	4.0
	Median (IQR)	14.00 (1.00)	13.00 (1.50) ***	9.00 (2.00) *** ###	7.00 (3.00) *** ### @@@	6.00 (2.00) *** ### @@@ $$$
	Maximum	15.0	15.0	12.0	10.0	10.0

The median total Manasabhava Scale (intensity) score at visit one (baseline visit; day zero) was 14, which gradually reduced to a median score of 6 at visit five (day 60±3). The total Manasabhava Scale (intensity) scores exhibited a significant reduction across all the visits, exhibiting a gradual reduction in the intensity of cognitive disorder domains throughout the assessment period of 60 days.

Although most of the domains (mano-vibhrama, bhakti-vibhrama, sheela-vibhrama, aachara-vibhrama, and swapna-viparyaya) of the Manasabhava Scale (intensity) scores also exhibited improvement, the statistical analysis for individual domains was not feasible due to the high number of ties and small sample size.

C. Changes in Total Manasabhava Scale (Frequency) Scores at Various Time Points

**Figure 4:**
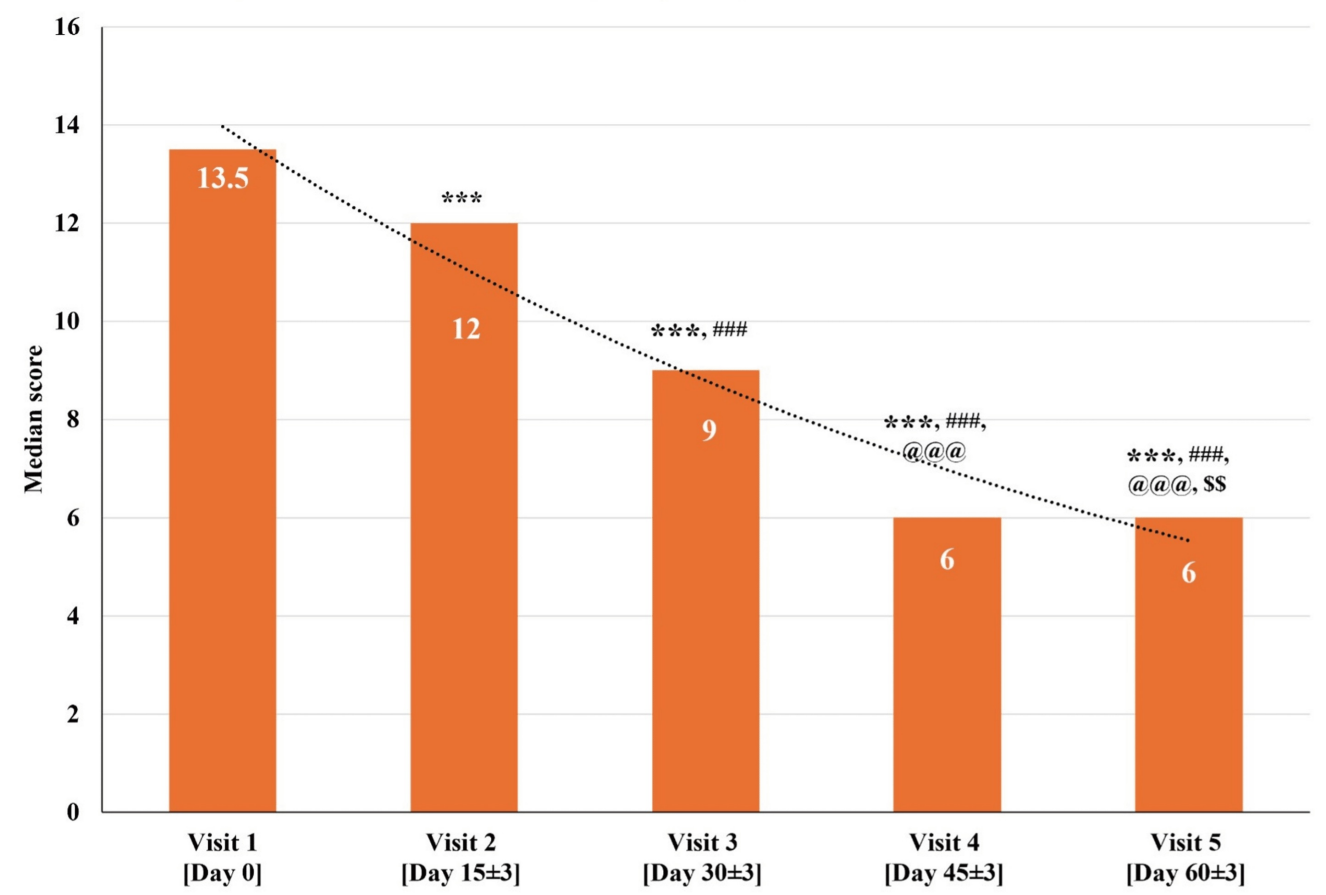
Changes in total Manasabhava Scale (Frequency) scores at various time points p<0.05 was considered significant. Results are expressed as median (IQR). Data were analyzed using the Wilcoxon signed-rank test. ^***^p<0.001 as compared to visit one (baseline; day zero),^ ###^p<0.001 as compared to visit two (day 15±3), ^@@@^p<0.001 as compared to visit three (day 30±3), and ^$$^p<0.01 as compared to visit four (day 45±3).

The total Manasabhava Scale (frequency) scores for all 30 participants at visit one (baseline visit; day zero) were between 8 and 16. The median total Manasabhava Scale (frequency) score significantly reduced (p<0.001) on visit two (day 15±3), visit three (day 30±3), visit four (day 45±3), and visit five (day 60±3) as compared to visit one (baseline visit; day zero). There was a significant reduction (p<0.001) in the median total Manasabhava Scale (frequency) score at visit three (day 30±3), visit four (day 45±3), and visit five (day 60±3) as compared to visit two (day 15±3). Further, there was a significant reduction (p<0.001) in the median total Manasabhava Scale (frequency) score at visit four (day 45±3), and visit five (day 60±3) as compared to visit three (day 30±3). Also, a significant reduction (p<0.01) was observed in the median total Manasabhava Scale (frequency) score at visit five (day 60±3) as compared to visit four (day 45±3) (Figure 4 and Table 5).

**Table 5:** Changes in the total Manasabhava Scale (frequency) scores at various time points p<0.05 was considered significant. Results are expressed as median (IQR). Data were analyzed using the Wilcoxon signed-rank test. ^***^p<0.001 as compared to visit one (baseline; day zero), ^###^p<0.001 as compared to visit two (day 15±3), ^@@@^p<0.001 as compared to visit three (day 30±3), and ^$$^p<0.01 as compared to visit four (day 45±3).

Parameter	Value	Visit one (V1) (baseline; day 0)	Visit two (V2) (day 15±3)	Visit three (V3) (day 30±3)	Visit four (V4) (day 45±3)	Visit five (V5) (day 60±3)
Total Manasabhava Scale (frequency) score	Minimum	8.0	6.0	6.0	4.0	4.0
	Median (IQR)	13.50 (3.00)	12.00 (3.25) ***	9.00 (3.00) *** ###	6.00 (4.00) *** ### @@@	6.00 (1.25) *** ### @@@ $$
	Maximum	16.0	15.0	12.0	10.0	10.0

The median total Manasabhava Scale (frequency) score at visit one (baseline visit; day zero) was 13.50, which gradually reduced to a median score of 6.00 at visit five (day 60±3). The total Manasabhava Scale (frequency) scores exhibited a significant reduction across all the visits, exhibiting a gradual reduction in the frequency of cognitive disorder domains throughout the assessment period of 60 days.

Although most of the domains (mano-vibhrama, bhakti-vibhrama, sheela-vibhrama, aachara-vibhrama, and swapna-viparyaya) of the Manasabhava Scale (frequency) scores also exhibited improvement, the statistical analysis for individual domains was not feasible due to the high number of ties and small sample size.

D. Changes in Total BCRS Scores at Various Time Points

At all five visits (visit one to visit five), for assessment of concentration, impairment of recent memory, impairment of past memory, orientation, and functioning and self-care, the BCRS was used [27,28].

**Figure 5:**
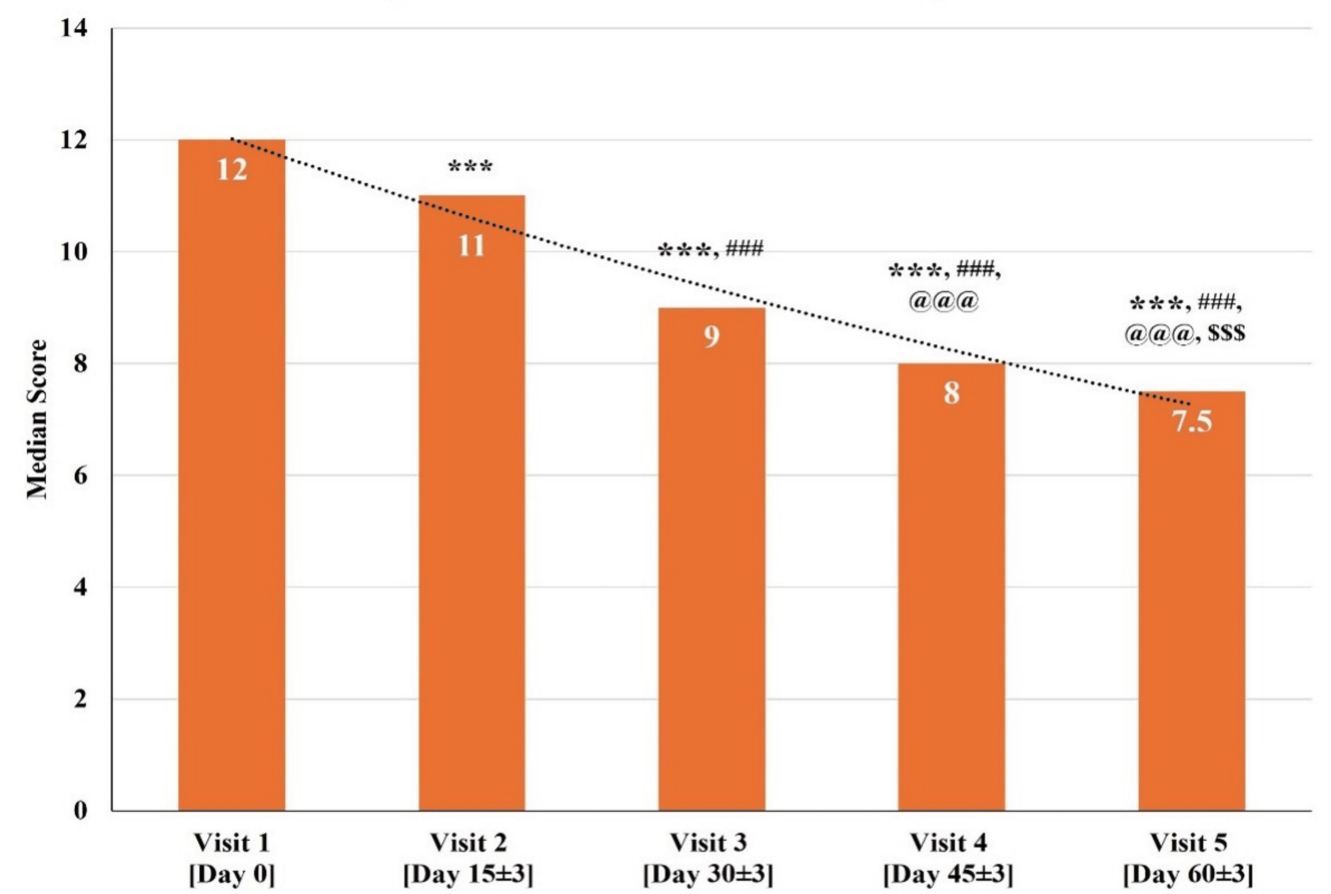
Changes in total Brief Cognitive Rating Scale (BCRS) scores at various time points p<0.05 was considered significant. Results are expressed as median (IQR). Data were analyzed using the Wilcoxon signed-rank test. ^***^p<0.001 as compared to visit one (baseline; day zero), ^###^p<0.001 as compared to visit two (day 15±3),^ @@@^p<0.001 as compared to visit three (day 30±3), and ^$$$^p<0.001 as compared to visit four (day 45±3).

The total BCRS scores for all 30 participants at visit one (baseline visit; day zero) were between 9 and 15. The median total BCRS score significantly reduced (p<0.001) on visit two (day 15±3), visit three (day 30±3), visit four (day 45±3), and visit five (day 60±3) as compared to visit one (baseline visit; day zero). There was a significant reduction (p<0.001) in the median total BCRS score at visit three (day 30±3), visit four (day 45±3), and visit five (day 60±3) as compared to visit two (day 15±3). Further, a significant reduction (p<0.001) in the median total BCRS score at visit four (day 45±3) and visit five (day 60±3) as compared to visit three (day 30±3) was observed. Also, a significant reduction (p<0.001) was observed in the median total BCRS score at visit five (day 60±3) as compared to visit four (day 45±3) (Figure 5 and Table 6).

**Table 6:** Changes in total Brief Cognitive Rating Scale (BCRS) scores at various time points p<0.05 was considered significant. Results are expressed as median (IQR). Data were analyzed using the Wilcoxon signed-rank test. ^***^p<0.001 as compared to visit one (baseline; day zero), ^###^p<0.001 as compared to visit two (day 15±3), ^@@@^p<0.001 as compared to visit three (day 30±3), and ^$$$^p<0.001 as compared to visit four (day 45±3).

Parameter	Value	Visit one (V1) (baseline; day zero)	Visit two (V2) (day 15±3)	Visit three (V3) (day 30±3)	Visit four (V4) (day 45±3)	Visit five (V5) (day 60±3)
Total BCRS score	Minimum	9.0	9.0	7.0	5.0	5.0
	Median (IQR)	12.00 (2.00)	11.00 (2.00) ***	9.00 (1.25) *** ###	8.00 (2.00) *** ### @@@	7.50 (1.25) *** ### @@@ $$$
	Maximum	15.0	13.0	13.0	10.0	9.0

The median total BCRS score at visit one (baseline visit; day zero) was 12.00, which gradually reduced to a median score of 7.50 at visit five (day 60±3). The total BCRS scores exhibited a significant reduction across all the visits, indicating a significant improvement in cognitive ability over a period of 60 days.

Although some domains (impairment of recent memory, impairment of past memory, and functioning and self-care) of BCRS also exhibited improvement, the statistical analysis for individual domains was not feasible due to the high number of ties and small sample size.

Secondary endpoints: changes in total scores of the CGI Scale, HAM-A, and PSQI at various time points

A. Changes in CGI Scale Scores at Various Time Points

The CGI Scale is a three-item observer-rated scale that measures illness severity (CGIS), global improvement or change (CGIC), and therapeutic response. [29-31] The median severity of illness scores on the CGI-1 scale remained relatively stable throughout the study period. It consistently remained at 2.00, indicating that most participants showed borderline illness (severity) across all visits. This suggests no worsening of mild NCD (severity of illness) among the participants throughout the study (Table 7).

**Table 7:** Changes in Clinical Global Impression (CGI) Scale scores at various time points

Parameter	Value	Visit one (V1) (baseline; day zero)	Visit two (V2) (day 15±3)	Visit 3 (V3) (day 30±3)	Visit four (V4) (day 45±3)	Visit five (V5) (day 60±3)
CGI-1: severity of illness	Minimum	2.0	1.0	1.0	1.0	1.0
	Median (IQR)	2.00 (1.00)	2.00 (1.00)	2.00 (0.00)	2.00 (1.00)	2.00 (1.00)
	Maximum	3.0	3.0	3.0	3.0	3.0
CGI-2: global improvement	Minimum	-	3.0	2.0	1.0	1.0
	Median (IQR)	-	4.00 (1.00)	3.00 (0.00)	2.00 (1.00)	2.00 (1.00)
	Maximum	-	4.0	4.0	4.0	4.0
CGI-3: efficacy index	Minimum	-	9.0	5.0	1.0	1.0
	Median (IQR)	-	9.00 (1.00)	9.00 (4.00)	5.00 (1.00)	5.00 (4.00)
	Maximum	-	13.0	9.0	9.0	5.0

According to the CGI-2 scale, participants exhibited a decrease in the median score for global improvement, with visit five (day 60±3) revealing a median score of 2.00, indicating a marked improvement compared to visit two (day 15±3). This sustained improvement suggests that Medhasagar Rasa treatment initiates positive changes and maintains effectiveness over time (Table 7).

The CGI-3 scale exhibited a steady improvement in the therapeutic effect of Medhasagar Rasa. Initially, participants experienced a slight improvement in their condition without any side effects. As treatment progressed, participants had a moderate therapeutic effect with partial remission of symptoms and no side effects. This suggests that Medhasagar Rasa treatment exhibits high efficacy and clinical safety, and its therapeutic effect becomes consistent and reliable over time, ensuring sustained improvement (Table 7).

B. Changes in total HAM-A Scores at Various Time Points

The HAM-A Scale is one of the first rating scales developed to measure the severity of anxiety symptoms and is still widely used today in both clinical and research settings. Since anxiety is one of the frequently reported neuropsychiatric symptoms in mild cognitive impairment, the HAM-A Scale was used in this study [32,33].

**Figure 6:**
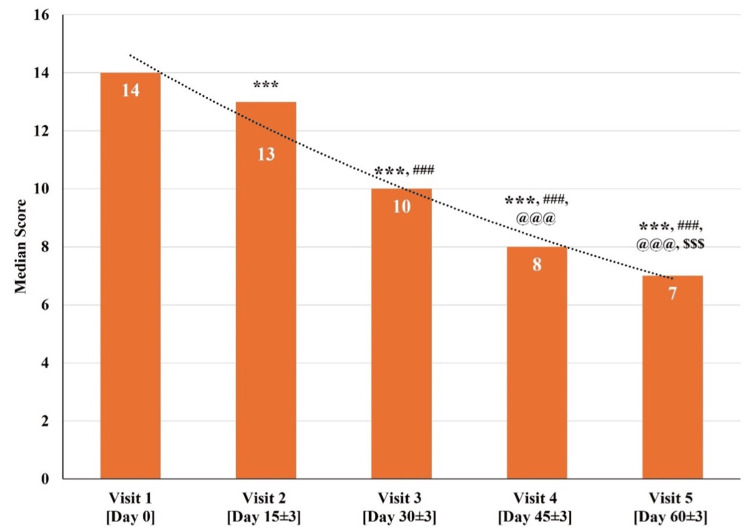
Changes in the total Hamilton Anxiety Rating Scale (HAM-A) scores at various time points p<0.05 was considered significant. Results are expressed as median (IQR). Data were analyzed using the Wilcoxon signed-rank test. ^***^p<0.001 as compared to visit one (baseline; day zero), ^###^p<0.001 as compared to visit two (day 15±3), ^@@@^p<0.001 as compared to visit three (day 30±3), and ^$$$^p<0.001 as compared to visit four (day 45±3).

The total HAM-A scores for all 30 participants at visit one (baseline visit; day zero) were between 11 and 19. The median total HAM-A score significantly reduced (p<0.001) on visit two (day 15±3), visit three (day 30±3), visit four (day 45±3), and visit five (day 60±3) as compared to visit one (baseline visit; day zero). There was a significant reduction (p<0.001) in the median total HAM-A Scale score at visit three (day 30±3), visit four (day 45±3), and visit five (day 60±3) as compared to visit two (day 15±3). Further, a significant reduction (p<0.001) in the median total HAM-A score at visit four (day 45±3) and visit five (day 60±3) as compared to visit three (day 30±3) was observed. Also, a significant reduction (p<0.001) was observed in the median total HAM-A score at visit five (day 60±3) as compared to visit four (day 45±3) (Figure 6 and Table 8).

**Table 8:** Changes in Total Hamilton Anxiety Rating Scale (HAM-A) scores at various time points p<0.05 was considered significant. Results are expressed as median (IQR). Data were analyzed using the Wilcoxon signed-rank test. ^***^p<0.001 as compared to visit one (baseline; day zero), ^###^p<0.001 as compared to visit two (day 15±3), ^@@@^p<0.001 as compared to visit three (day 30±3), and ^$$$^ p<0.001 as compared to visit four (day 45±3).

Parameter	Value	Visit one (V1) (baseline; day zero)	Visit two (V2) (day 15±3)	Visit three (V3) (day 30±3)	Visit four (V4) (day 45±3)	Visit five (V5) (day 60±3)
Total HAM-A score	Minimum	11.0	10.0	8.0	6.0	5.0
	Median (IQR)	14.00 (2.00)	13.00 (2.00) ***	10.00 (2.00) *** ###	8.00 (2.00) *** ### @@@	7.00 (1.00) *** ### @@@ $$$
	Maximum	19.0	16.0	12.0	10.0	9.0

The median total HAM-A Scale score at visit one (baseline visit; day zero) was 14, which gradually reduced to a median score of 7 at visit five (day 60±3). The total HAM-A score exhibited a significant reduction across all the visits, indicating a significant reduction in anxiety levels over a period of 60 days.

Although many domains (anxious mood, tension, insomnia, and depressed mood) of the HAM-A Scale also exhibited improvement, the statistical analysis for individual domains was not feasible due to the high number of ties and small sample size.

C. Changes in Total PSQI Scale Scores at Various Time Points

PSQI Scale is a self-rated questionnaire to assess sleep quality and disturbances. Since sleep disturbance is one of the frequently reported neuropsychiatric symptoms in MCI, the PSQI Scale was used in this study [33,34].

**Figure 7:**
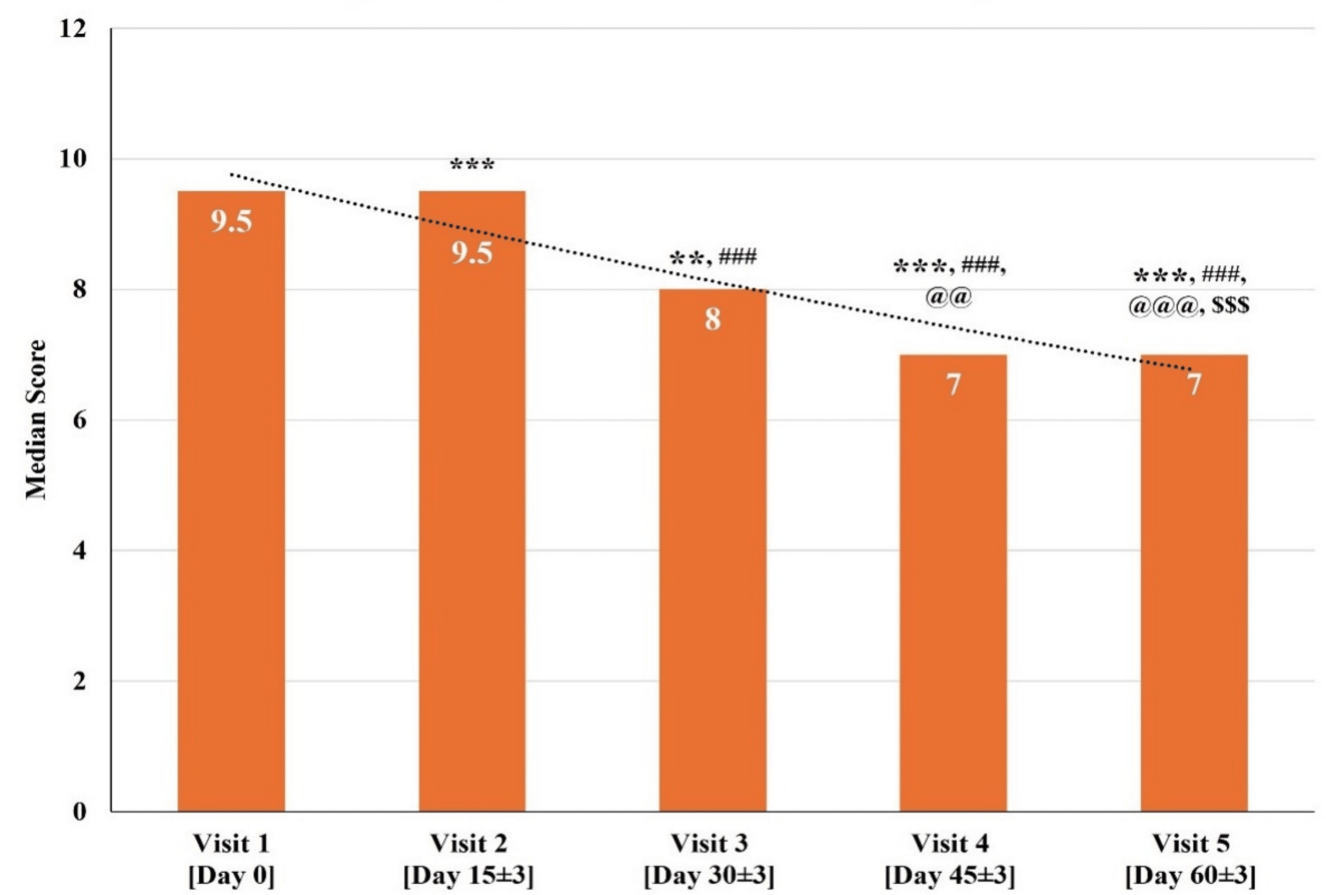
Changes in total Pittsburg Sleep Quality Index (PSQI) Scale scores at various time points p<0.05 was considered significant. Results are expressed as median (IQR). Data were analyzed using the Wilcoxon signed-rank test. ^***^p<0.001 & ^**^p<0.01 as compared to visit one (baseline; day zero), ^###^p<0.001 as compared to visit two (day 15±3), ^@@@^p<0.001, ^@@^p<0.01 as compared to visit three (day 30±3), and ^$$$ ^p<0.001 as compared to visit four (day 45±3).

The total PSQI Scale scores for all 30 participants at visit one (baseline visit; day zero) were between 7 and 18. The median total PSQI Scale score significantly reduced (p<0.001, p<0.01, p<0.001, p<0.001) on visit two (day 15±3), visit three (day 30±3), visit four (day 45±3), and visit five (day 60±3). There was a significant reduction (p<0.001) in the median total PSQI Scale score at visit three (day 30±3), visit four (day 45±3), and visit five (day 60±3) as compared to visit two (day 15±3). Further, a significant reduction (p<0.01, p<0.001) in the median total PSQI Scale score at visit four (day 45±3) and visit five (day 60±3) as compared to visit three (day 30±3) was observed. Also, a significant reduction (p<0.001) was observed in the median total PSQI Scale score at visit five (day 60±3) as compared to visit four (day 45±3) (Figure 7 and Table 9).

**Table 9:** Changes in Total Pittsburg Sleep Quality Index (PSQI) Scale scores at various time points p<0.05 was considered significant. Results are expressed as median (IQR). Data were analyzed using the Wilcoxon signed-rank test.^ ***^p<0.001, ^**^p<0.01 as compared to visit one (baseline; day zero), ^###^p<0.001 as compared to visit two (day 15±3), ^@@@^p<0.001, ^@@^p<0.01 as compared to visit three (day 30±3), and ^$$$^p<0.001 as compared to visit four (day 45±3).

Parameter	Value	Visit one (V1) (baseline; day zero)	Visit two (V2) (day 15±3)	Visit three (V3) (day 30±3)	Visit four (V4) (day 45±3)	Visit five (V5) (day 60±3)
Total PSQI Scale score	Minimum	7.0	6.0	3.0	3.0	3.0
	Median (IQR)	9.50 (3.25)	9.50 (3.00) ***	8.00 (2.00) ** ###	7.00 (2.00) *** ### @@	7.00 (3.00) *** ### @@@ $$$
	Maximum	18.0	17.0	16.0	16.0	14.0

The median total PSQI Scale score at visit one (baseline visit; day zero) was 9.50, which gradually decreased to a median score of 7.00 at visit five (day 60±3). The total PSQI Scale scores exhibited a significant reduction across all the visits, indicating a significant improvement in sleep quality over a period of 60 days.

Assessment of safety at various time points

A. Number of Participants Showing Adverse Events at Various Time Points

No serious adverse events were reported/observed at all time points of measurement, i.e., visit one (baseline visit; day zero), visit two (day 15±3), visit three (day 30±3), visit four (day 45±3), and visit five (day 60±3) in any of the participants.

B. Changes in Vital Parameters (Temperature, Blood Pressure, Pulse Rate, and Respiratory Rate) at Various Points

All vital parameters, i.e., temperature, blood pressure, heart rate, and respiratory rate were found to be within normal ranges, at all-time points of measurement. No statistically significant change was observed in any vital parameter of the 30 participants, measured on visit one (baseline visit; day zero), visit two (day 15±3), visit three (day 30±3), visit four (day 45±3), and visit five (day 60±3).

C. Changes in Systemic Examination Parameters at Various Points

All systemic examination variables (cardiovascular system (CVS), central nervous system (CNS), respiratory system (RS), and per abdomen) were found to be normal at all time points of measurement. No statistically significant change was observed in any systemic examination of the 30 participants, done on visit one (baseline visit; day zero), visit two (day 15±3), visit three (day 30±3), visit four (day 45±3), and visit five (day 60±3). 

D. Changes in Laboratory Investigations (SGOT, SGPT, BUN, Creatinine, FBS, and Postprandial Blood Sugar (PPBS) Done at Visit One (Baseline Visit; Day Zero) and Visit Five (Day 60±3)

No statistically significant changes were found in the median SGOT, SGPT, BUN, Creatinine, FBS, and PPBS values at visit five (day 60±3) when compared to visit one (baseline visit; day zero) values (Table 10).

**Table 10:** Changes in laboratory investigations (SGOT, SGPT, BUN, Creatinine, FBS, and PPBS) done at visit one (baseline visit; day zero) and visit five (day 60±3) p<0.05 was considered significant. Results are expressed as median (IQR). Data were analyzed using the Wilcoxon signed-rank test. NS: not significant as compared with visit one (baseline; day zero).

Laboratory Investigation	Value	Visit one (V1) (baseline; day zero)	Visit five (V5) (day 60±3)
Serum glutamic-oxaloacetic transaminase (SGOT) (U/L)	Minimum	16.0	16.0
	Median (IQR)	22.50 (5.5)	23.00^NS ^(7.0)
	Maximum	36.0	36.0
Serum glutamic pyruvic transaminase (SGPT) (U/L)	Minimum	16.0	15.0
	Median (IQR)	22.00 (7.0)	22.00^NS ^(7.0)
	Maximum	39.00	31.00
Serum blood urea nitrogen (BUN) (mg/dL)	Minimum	8.4	8.4
	Median (IQR)	10.73 (2.92)	10.73^NS ^(2.8)
	Maximum	17.7	16.8
Serum creatinine (mg/dL)	Minimum	0.7	0.8
	Median (IQR)	0.90 (0.175)	0.90^NS ^(0.1)
	Maximum	1.1	1.3
Fasting blood sugar (FBS) (mg/dL)	Minimum	75.0	79.0
	Median (IQR)	92.50 (19.0)	96.00^NS ^(11.5)
	Maximum	134.0	140.0
Postprandial blood sugar (PPBS) (mg/dL)	Minimum	89.0	89.0
	Median (IQR)	113.00 (33.75)	115.00^NS ^(9.25)
	Maximum	153.0	176.0

Post-study power analysis of the primary endpoint

The power of the study was calculated based on the observed improvement in the total MoCA Scale scores. The effect size was determined by dividing the difference between the final median MoCA score (25) and the baseline median MoCA score (20) by the estimated standard deviation (5), yielding an effect size of 1.0. Using this effect size, along with a sample size of 30 participants and a significance level (α) of 0.05, a power analysis was performed, resulting in a power of approximately 99.96%, indicating a very high likelihood of detecting a statistically significant difference in cognitive function if such a difference exists.

## Discussion

An NCD represents a transitional stage between normal aging and dementia, characterized by a decline in cognitive abilities that exceeds typical age-related changes but is not severe enough to warrant a diagnosis of dementia [1,35]. Individuals with this disorder experience difficulties with memory, language, and executive function. The annual rates of progression to dementia were estimated to range from 5-10% with higher estimates in clinical versus community samples. Risk factors for mild NCDs include advanced age, medical conditions such as diabetes, depression, stroke, genetic predispositions, and lifestyle factors [36]. Early detection and intervention are crucial for implementing strategies to slow cognitive decline, improve cognitive function, and enhance overall quality of life [37].

Currently, there is no strong evidence that supports interventions for cognition support in MCI [38]. Certain drugs, such as cholinesterase inhibitors like donepezil, rivastigmine, and galantamine, may alleviate symptoms but their use in MCI was not associated with any delay in the onset of Alzheimer's disease (AD) or dementia [39]. Additionally, cholinesterase inhibitors can increase the availability of acetylcholine, causing overstimulation of the parasympathetic nervous system, leading to SLUDGE (salivation, lacrimation, urination, diaphoresis, gastrointestinal upset, and emesis) syndrome [40]. Therefore, it is necessary to update the search for evidence concerning the treatment of mild NCDs, emphasizing the importance of considering conventional, complementary, and alternative therapies [6].

In this exploratory study, the effectiveness of Medhasagar Rasa was evaluated in participants of either sex between 50-70 years of age suffering from age-associated mild NCDs. Assessing the severity of symptoms in age-associated NCDs is challenging due to the subjective nature of the condition. A diverse range of symptoms further complicates this, making objective measurement difficult. Additionally, there needs to be more consensus on assessment methods, resulting in the use of various tools and scales. In this study, the effectiveness of Medhasagar Rasa on cognitive and functional abilities and quality of life in participants with age-associated mild NCDs was assessed by using the MoCA, BCRS, and Manasabhava Scales [24-28]. Furthermore, the effect of Medhasagar Rasa on physician’s global assessment of illness, anxiety levels, and sleep quality was evaluated using the CGI Scale, HAM-A, and PSQI, respectively. Collectively, these scales were used to gain comprehensive insights into various aspects of cognitive health, including cognitive functions, anxiety levels, and sleep quality, all of which are intricately linked to cognitive decline [29-34].

In this study, NCD diagnosis and assessment were done based on the MoCA Scale score. The MoCA Scale scores of 26 or above are considered normal and scores of 25 or below indicate impairment [24,25]. A gradual increase in the total median MoCA Scale score from 20 to 25 was reported over a treatment period of 60 days (Figure 2 and Table 3). This consistent improvement in MoCA Scale score at all follow-up visits compared to the baseline visit indicates an overall enhancement in cognitive abilities, with participants transitioning from MCI to improved cognitive functioning during the study period.

Additionally, to support the findings of the MoCA Scale, the BCRS was used. The BCRS provides a more specific assessment of cognitive functions. The BCRS yields valuable information regarding the progression of cognitive decline and the impact of such decline on behavior and function [27,28]. Medhasagar Rasa treatment exhibited a gradual significant reduction in the total median BCRS Scale score from 12 to 7.5 over a treatment period of 60 days indicating enhanced cognitive abilities in the study participants (Figure 5 and Table 6).

A common neuropsychiatric symptom in MCI cases is anxiety, which is reported to occur in 26.3% of clinical samples and roughly 11.6% of population-based samples [33]. Furthermore, a longitudinal study of 368 adults aged more than 65 years from a population-based cohort confirmed a significant bidirectional association between anxiety and MCI [41]. Several studies have demonstrated that the presence of anxiety may accelerate the progression from MCI to dementia [42,43]. In a study tracking MCI patients for up to four years, those who exhibited anxiety, as measured by the Hospital Anxiety and Depression Scale, showed a correlation with the deposition of amyloid-β (Aβ) [44]. Furthermore, anxiety predicted cognitive decline and interacted with amyloid status, leading to faster cognitive deterioration, as confirmed by numerous investigations associating anxiety with positive amyloid scans [44,45]. In this study, the anxiety score was assessed by using the HAM-A. There was a consistent and significant (p<0.001) decrease in total median HAM-A scores from 14 to 7 over a period of 60 days, indicating a significant reduction in anxiety symptoms (from mild-moderate anxiety to mild anxiety) and positive therapeutic effects of Medhasagar Rasa on mild NCD-associated anxiety (Figure 6 and Table 8).

In elderly individuals, a cross-sectional study demonstrated that those with depression and mild NCDs exhibited lower quality of sleep compared to healthy controls, suggesting a potential association between sleep quality, quality of life, and cognitive function [46]. In this study, the median total PSQI Scale score exhibited a significant (p<0.001) reduction from 9.5 to 7 over a period of 60 days. This consistent decrease in the PSQI Scale score indicates a positive impact of Medhasagar Rasa on sleep disturbances associated with mild NCDs (Figure 7 and Table 9).

In conjunction with the MoCA, BCRS, HAM-A, and PSQI scales, the Manasabhava Scale was also adopted to assess Ayurved domains linked to cognitive impairment [26]. The total Manasabhava Scale scores exhibited a significant reduction in intensity (median score decreased from 14 to 6) and frequency (median score decreased from 13.50 to 6) in the study participants (Figure 3-4 and Table 4-5). This improvement in the cognitive domains of the Manasabhava Scale aligned with other validated scales such as MoCA, BCRS, HAM-A, and PSQI scales.

Furthermore, the effectiveness of Medhasagar Rasa was also assessed by means of the CGI Scale. The CGI scale was developed for clinical trials to concisely evaluate a patient's global functioning before and after starting a study medication. Over 30 years, the CGI has shown a strong correlation with established research drug efficacy scales such as HAM-Depression, HAM-A, BCRS, and others [29-31]. In this study, the CGI-1 domain (severity of illness) exhibited stable median scores at 2.00, suggesting no worsening. The CGI-2 domain (global improvement) exhibited a decreasing trend indicating a significant improvement in participants' condition by visit five (day 60±3) as compared to visit two (day 15±3), with sustained improvement. The CGI-3 domain (efficacy index) demonstrated an improvement in the therapeutic effect by visit two (day 15±3), which became more consistent by visit four (day 45±3) and visit five (day 60±3), suggesting a better and consistent effect of Medhasagar Rasa treatment without side effects (Table 7). Based on the findings, all participants demonstrated significant improvement in cognitive function across the measured scales, with no individual cases explicitly identified as non-responders or showing minimal improvements for the primary and secondary endpoints.

Many studies have shown that cognitive decline in the human brain, encompassing memory loss, reduced concentration, and orientation difficulties, arises from several molecular and cellular pathways such as oxidative stress, inflammation, dysregulation of neuronal calcium homeostasis, mitochondrial dysfunction, deregulated autophagy, alterations in energy metabolism, changes in brain structure, and neurodegenerative disorders [47-49]. In this study, the improvement in cognitive ability, anxiety reduction, and improvement of sleep in participants suffering from mild NCD can be attributed to the Medhya (nootropic) action of herbomineral, mineral, and herbal constituents in Medhasagar Rasa.

The herbomineral and mineral constituents in Medhasagar Rasa are Mahakalyan Vati, Smrutisagar Rasa, Rajata Bhasma (processed silver), and Abhraka Bhasma (processed mica). The individual ingredients of Mahakalyan Vati are documented for their beneficial role in improving cognitive functions [11]. Suvarna Bhasma (processed gold), one of the prime constituents in Mahakalyan Vati, improves cognitive functions and is documented for its anxiolytic effects and neuroprotective actions in animal models [19,20]. Rajata Bhasma (processed silver) and Abhraka Bhasma (processed mica) are Medhya (nootropic) formulations used singly or in combination [13,14]. Smrutisagar Rasa, another classical herbomineral component in Medhasagar Rasa is widely used by Ayurved practitioners to improve cognitive functions [12]. The neuroprotective actions of herbal components of Smrutisagar Rasa, viz., Vacha (*Acorus calamus*), Brahmi (*Bacopa monnieri*), and Jyotishmati (*Celastrus paniculatus*) help in memory preservation, reduce oxidative stress, and prevent deposition of amyloid-β plaque in the brain [21-23].

Furthermore, the herbs such as Mandookaparni (*Centella asiatica*), Shankhapushpi (*Convolvulus pluricaulis*), Yashti (*Glycyrrhiza glabra*), and Guduchi (*Tinospora cordifolia*) in Medhasagar Rasa possess memory-enhancing, nootropic, and neuroprotective properties [16, 50-55]. Ashvagandha (*Withania somnifera*) and Haridra (*Curcuma longa*) are reported to prevent amyloid-β plaque build-up in the brain [17,18]. Ashvagandha (*Withania somnifera*) has also exhibited a neuroprotective role in neurodegenerative diseases (Alzheimer's, Huntington's, and Parkinson's) by restoring mitochondrial and endothelial functions and mitigating apoptosis, inflammation, and oxidative stress mechanisms [56]. In addition to its anxiolytic and anti-stress actions, Ashvagandha (*Withania somnifera*) has also reported beneficial effects in reducing the time to fall asleep, enhancing sleep duration and quality, and positively influencing mood upon awakening [57,58].

Shankhapushpi (*Convolvulus pluricaulis*) has demonstrated neuropharmacological effects, including memory enhancement, anti-depressant, and anxiolytic properties, particularly targeting serotonergic synapses crucial for anxiety regulation [59]. The significant improvements in MoCA scores, anxiety levels (HAM-A), and sleep quality (PSQI) throughout the study demonstrate Medhasagar Rasa's efficacy in addressing the multifaceted challenges of mild NCDs. Its blend of herbomineral and herbal ingredients with documented neuroprotective and cognitive-enhancing properties provides a promising alternative or adjunctive treatment for patients with MCI, supporting its potential for wider clinical adoption. Integrating Medhasagar Rasa into treatment protocols could offer a more holistic option to enhance cognitive outcomes in aging populations.

No adverse events were reported or observed among participants taking Medhasagar Rasa. Vital parameters remained consistently within the normal range during all time points throughout the course of the study. No statistically significant changes were found in the median SGOT, SGPT, BUN, Creatinine, FBS, and PPBS values at visit five (day 60±3) when compared to visit one (baseline visit; day zero) values (Table 10). These findings imply that the participants maintained relatively stable liver and kidney functions from baseline to final visit. The compliance and adherence in taking study medication was well within the acceptable range (≥80%).

Despite certain limitations, such as a single-arm, single-center design, a smaller sample size, and a shorter intervention duration of 60 days, this exploratory clinical trial offers valuable insights into the cognitive enhancement potential of Medhasagar Rasa. A post-study power analysis revealed a power of approximately 99.96%, indicating a high likelihood of detecting a significant effect if present, which strengthens confidence in the outcomes. However, the small sample size highlights the need for future studies with larger, more diverse populations to confirm these findings. To further enhance the validity and applicability of the results, future research should incorporate a randomized, double-arm control trial framework and a multicentric approach to improve generalizability. Extending the intervention period beyond 60 days would also provide more comprehensive data on the long-term effects of Medhasagar Rasa. These refinements would bolster the study's clinical relevance and support the wider adoption of Medhasagar Rasa in cognitive health management.

## Conclusions

The findings of this clinical trial demonstrate the effectiveness of Medhasagar Rasa in managing age-associated mild NCDs, with significant improvements in cognitive functions, anxiety, and sleep disturbances. Medhasagar Rasa presents a promising, safe, and comprehensive therapeutic option in a field with limited evidence-based treatments. The improvements in MoCA, BCRS, HAM-A, and PSQI scores highlight its potential to address cognitive decline and related symptoms. However, limitations such as the single-arm, single-center design, small sample size, and short intervention period necessitate careful consideration of the results. While a post-study power analysis confirmed a high power, further large-scale, randomized, double-arm studies with extended intervention periods and a multicentric approach are needed to validate these findings and explore the long-term efficacy of Medhasagar Rasa. These future studies will help establish its broader clinical relevance and support its wider adoption in cognitive outcomes.
